# On Disease Modifying and Neuroprotective Treatments for Parkinson's Disease: Physical Exercise

**DOI:** 10.3389/fneur.2022.938686

**Published:** 2022-07-14

**Authors:** Pedro J. Garcia Ruiz, Rosario Luquin Piudo, Juan Carlos Martinez Castrillo

**Affiliations:** ^1^Department of Neurology, Fundación Jiménez Díaz, Madrid, Spain; ^2^Department of Neurology, Clinica Universidad de Navarra, Pamplona, Spain; ^3^Department of Neurology, Hospital Ramón y Cajal, Madrid, Spain

**Keywords:** Parkinson's disease, disease modifying agents, neuroprotection, physical activity, preventive treatment

## Introduction

For decades, the concept of “disease-modifying” treatment has been used profusely in several neurodegenerative diseases including Parkinson's disease (PD). This concept has been employed sometimes as an accomplished goal, and many more as a wishful thinking objective. In addition, the related concept of neuroprotective therapy has been widely used and a quick search on Medline shows a steady increase in the number of papers on this issue over time.

As Morant et al. (1) pointed out there is a particular interest in conceptually distinguishing disease-modifying treatments from symptomatic-only treatments.

Perhaps it is time to ponder over these two related concepts: disease modifying and neuroprotective therapies, at least in relation to PD. First, it is important to have (more or less) clear definitions, and then, we can discuss whether we have or we may have (or not) disease modifying and neuroprotective therapy for PD.

To begin with, the definition of “disease-modifying” treatment varies both within and between neurodegenerative disorders, and terminology in current regulatory guidelines also lacks consistency (1). Cummings suggested that disease modification can be defined as treatments or interventions that affect the underlying pathophysiology and have beneficial outcome on the course of the disease (2). Since the pathophysiology of PD is only partially known, we can employ a more clinical approach for disease-modifying measures as “effective treatments that modify the course of PD and maintain or improve patient quality of life” (3).

Here it must be pointed out that, in theory, any disease-modifying treatment may have symptomatic effect as well, possibly masking the modifications produced in the disease. Recently, Vijiaratnam et al. (3) reviewed the crucial issue of why we have failed to demonstrate disease-modifying effect of treatments on PD. Several reason may partly explain this shortcoming, including the complex pathophysiology and heterogeneity of the disease (3), but from a clinical viewpoint, detecting real modification with any given treatment may take an extended period of time.

To date, no disease-modifying drugs has been found, although some promising candidates are still in the pipeline including exenatide and gene therapy (4).

And still, probably, a real disease-modifying treatment for PD already exists and has been used for decades: Physical exercise. As Eric Ahlskog already suggested a decade ago, “often overlooked (…) is the potential benefit of sustained vigorous exercise on PD progression” (5).

In this short review, we collect and summarize, the most relevant data of physical exercise as a symptomatic and preventive measure, and its potential for disease modifying therapy for patients with PD.

## Physical Exercise as Symptomatic Treatment

Although the role of physical exercise as symptomatic treatment for PD was already suggested in classic texts (6), this non-pharmacological approach attracted renew interest in the 90s; Comella et al. (7) carried out a controlled trial testing physical exercise in a group of moderately advanced PD. They authors found statistically significant difference in the experimental group, but also observed that motor improvement was not sustained once normal activity was resumed (7). Many other clinical trials, reviews and meta-analyses have been published on this issue over the last decades (5, 8–14). One particularly noteworthy example is the randomized controlled trial by Corcos et al. (8) which showed that progressive resistance exercise demonstrated a statistically significant reduction in UPDRS-III scores (8). Schenkman et al. (13) in a recent randomized clinical trial, studied the effect of high-intensity endurance exercise on motor symptoms in *de novo* PD patients. They found statistical differences in Unified Parkinson's Disease Rating Scale (UPSRS) motor score in the high-intensity group compared with the usual care group. Another interesting study was carried out by van der Kolk et al. (14). In a double blind randomized controlled trial, the authors studied the effectiveness of home-based supervised aerobic exercise on PD; The off-state MDS-UPDRS motor score revealed a significant difference in favor of aerobic exercise.

In addition, physical exercise showed potential to increase the efficacy of antiparkinsonian medication (9, 11). Recently, in their excellent overview of physical exercise on PD; Mak et al. suggested that exercise training can modify long term motor symptoms in PD (11). Finally, da Silva et al. (10) carried out a systematic review of physical exercise on cognitive function of PD; they suggested that physical exercise promotes significant effects on global cognitive function, processing speed, sustained attention and mental flexibility in PD patients. Even when used only as symptomatic treatment, physical exercise should be widely considered as a fundamental antiparkinsonian measure (5, 8–14).

## Physical Exercise as a Disease-Modifying Treatment

In addition to having confirmed symptomatic antiparkinsonian effect, physical exercise may attenuate and influence the natural history of PD (5, 15–19). An increasing evidence suggests that vigorous exercise may exert a disease-modifying effect on PD (5, 15). A very recent publication from Japan showed that the maintenance of high physical exercise was clearly associated with better clinical course of PD (16). To date, the published evidence is indirect and based mainly on observational cohort studies and/or meta-analyzes (16–18), but it is worth stressing the inverse dose-response association between the amount of physical exercise with evolution and mortality in PD (17, 18).

## Physical Exercise as a Preventive Measure

If any disease-modifying treatment exits, then it would most likely be useful also as a preventive measure for neurodegenerative diseases including PD. Epidemiologic evidence suggests that physical exercise may protect against PD (20–25). Habitual vigorous exercise in midlife reduced the risk of later-developing PD in several cohorts (20–25). Physical exercise also seems to confer protective effect on different neurodegenerative diseases such as PD, Alzheimers's disease, Huntington's disease and degenerative ataxias (26). According to this data, exercise training would be a practical and inexpensive guide to counsel patients at risk of PD, such as LRRK2 carriers and others.

## Exercise: Mechanisms of Action

Physical exercise has been recommended since the times of Hippocrates and Galen as a general measure for health and disease prevention (27), but its mechanism has been completely unknown for centuries. At present, some of the potential mechanisms have been studied both in experimental animal models (28–30), and in patients with neurodegenerative diseases as well as controls (31–34).

Potential mechanisms include neuronal survival and plasticity, neurogenesis, epigenetic modifications, angiogenesis, autophagy, and the synthesis and release of neurotrophins (28–35).

Possibly, the most interesting and testable mechanism includes the release of Brain Derived Neurotrophic Factor (BDNF) (28–34), suffice is to recall that BDNF is a crucial neurotrophic factor with multiple roles on regulation of neurophysiological processes (35), including survival of striatal neurons (36). Physical exercise increases plasma BDNF levels in individuals with neurodegenerative disorders (34); and interestingly, BDNF receptor blockade prevents the beneficial effects of exercise in animal models (29).

## Conclusion

If physical exercise is symptomatically effective, probably prevents neurodegenerative diseases, and has potential neuroprotective mechanisms, why is it not universally used?

This conundrum may be explained by several factors. There are barriers to exercise (37, 38), as Ellis et al. suggested, low outcome expectation from exercise, lack of time, and fear of falling appear to be important perceived barriers to exercise. In addition, optimal benefit requires active and sustained participation of patients and families (37, 38). Finally, as Alberts commented (12), frequently, exercise recommendations lack specificity in terms of frequency, intensity and duration.

In summary, although physical exercise is inexpensive, its use as a treatment requires a complete change of strategy. Exercise training would be considered the first antiparkinsonian measure, even before (or at least at the same time) than drug therapy is added (12, 38, 39). Changes are not easy to implement, although other medical specialties such as endocrinology have well-designed patient education programs for chronic diseases uch as diabetes. Certainly, a long-term prospective studies are needed to confirm the neuroprotective capacity of physical exercise on PD (40). Ongoing Clinical Trials, (Including SPARX3 and CYCLE-II) Have Potential to Further Develop Patient-Specific Exercise Recommendations (12). Confirming this effect of exercise training would revolutionize the way we treat patients with neurodegenerative diseases, and also would open new avenues of basic and clinical research.

Finally, physical exercise would be a practical and inexpensive approach for those patients at risk for PD (such as LRKK2 carriers).

## Author Contributions

PG conception and design, interpretation of data, drafting the submitted material, and critical review. RL and JM drafting the submitted material and critical review. All authors contributed to the article and approved the submitted version.

## Conflict of Interest

PG received research support from Allergan and UCB, personal compensation as a consultant/scientific advisory board from Italfarmaco, Britannia, Bial, and Zambon and speaking honoraria from Italfarmaco, Zambon, Allergan, and Abbvie. JM has received honoraria as a speaker from AbbVie, Allergan, Bial, Krka, Merz, Ipsen, Italfarmaco, Medtronic, TEVA, and Zambon; travel grants from AbbVie, Allergan, Bial, Italfarmaco, TEVA, Merz, Krka and Zambón; research grants from AbbVie, Allergan, Merz, Italfarmaco, and Zambon; and participated in advisory boards of AbbVie, Allergan, Bial, Merz, Italfarmaco, Lundbeck, Orion, UCB, and Zambon. RL has received honoraria for lecturing and advisory board from AbbVie, Lundbeck, UCB, and Italfarmaco and research grants from the European Commission and Instituto de Salud Carlos III.

## Publisher's Note

All claims expressed in this article are solely those of the authors and do not necessarily represent those of their affiliated organizations, or those of the publisher, the editors and the reviewers. Any product that may be evaluated in this article, or claim that may be made by its manufacturer, is not guaranteed or endorsed by the publisher.

## References

[1] Morant AV Jagalski V Vestergaard HT. Labeling of disease-modifying therapies for neurodegenerative disorders. Front Med (Lausanne). (2019) 6:223. 10.3389/fmed.2019.00223 31681780 PMC6811601

[2] Jeffrey L. Cummings defining and labeling disease-modifying treatments for Alzheimer's disease. Alzheimers Dement. (2009). 5:406–18. 10.1016/j.jalz.2008.12.003 19751920

[3] Vijiaratnam N Simuni T Bandmann O Morris HR Foltynie T. Progress towards therapies for disease modification in Parkinson's disease. Lancet Neurol. (2021) 20:559–72. 10.1016/S1474-4422(21)00061-2 34146514

[4] McFarthing K Rafaloff G Baptista MAS Wyse RK Stott SRW. Parkinson's disease drug therapies in the clinical trial pipeline: 2021 update. J Parkinsons Dis. (2021) 11:891–903. 10.3233/JPD-219006 34151864 PMC8461678

[5] Ahlskog JE. Does vigorous exercise have a neuroprotective effect in Parkinson disease? Neurology. (2011) 77:288–94. 10.1212/WNL.0b013e318225ab66 21768599 PMC3136051

[6] Wilson SAK. Paralysis agitans. In: Bruce N, editor. Neurology by Kinnier Wilson, Vol. II. London: Edward Arnold & Co (1940). p. 787–804.

[7] Comella CL Stebbins GT Brown-Toms N Goetz CG. Physical therapy and Parkinson's disease: a controlled clinical trial. Neurology. (1994) 44(3 Pt 1):376–8. 10.1212/WNL.44.3_Part_1.376 8145901

[8] Corcos DM Robichaud JA David FJ Leurgans SE Vaillancourt DE Poon C. A two-year randomized controlled trial of progressive resistance exercise for Parkinson's disease. Mov Disord. (2013) 28:1230–40. 10.1002/mds.25380 23536417 PMC3701730

[9] Ferrazzoli D Ortelli P Riboldazzi G Maestri R Frazzitta G. Effectiveness of Rotigotine plus intensive and goal-based rehabilitation versus Rotigotine alone in “de-novo” Parkinsonian subjects: a randomized controlled trial with 18-month follow-up. J Neurol. (2018) 265:906–16. 10.1007/s00415-018-8792-0 29442177 PMC5878188

[10] da Silva FC Iop RDR de Oliveira LC Boll AM de Alvarenga JGS Gutierres Filho PJB. Effects of physical exercise programs on cognitive function in Parkinson's disease patients: a systematic review of randomized controlled trials of the last 10 years. PLoS ONE. (2018) 13:e0193113. 10.1371/journal.pone.0193113 29486000 PMC5828448

[11] Mak MK Wong-Yu IS Shen X Chung CL. Long-term effects of exercise and physical therapy in people with Parkinson disease. Nat Rev Neurol. (2017) 13:689–703. 10.1038/nrneurol.2017.128 29027544

[12] Alberts JL Rosenfeldt AB. The universal prescription for Parkinson's disease: exercise. J Parkinsons Dis. (2020) 10:S21–7. 10.3233/JPD-202100 32925109 PMC7592674

[13] Schenkman M Moore CG Kohrt WM Hall DA Delitto A Comella CL. Effect of high-intensity treadmill exercise on motor symptoms in patients with de novo parkinson disease: a phase 2 randomized clinical trial. JAMA Neurol. (2018) 75:219–26. 10.1001/jamaneurol.2017.3517 29228079 PMC5838616

[14] van der Kolk NM de Vries NM Kessels RPC Joosten H Zwinderman AH Post B. Effectiveness of home-based and remotely supervised aerobic exercise in Parkinson's disease: a double-blind, randomized controlled trial. Lancet Neurol. (2019) 18:998–1008. 10.1016/S1474-4422(19)30285-6 31521532

[15] Ahlskog JE. Aerobic exercise: evidence for a direct brain effect to slow Parkinson disease progression. Mayo Clin Proc. (2018) 93:360–72. 10.1016/j.mayocp.2017.12.015 29502566

[16] Tsukita K Sakamaki-Tsukita H Takahashi R. Long-term effect of regular physical activity and exercise habits in patients with early Parkinson disease. Neurology. (2022) 98:e859–1. 10.1212/WNL.0000000000013218 35022304 PMC8883509

[17] Merola A Romagnolo A Dwivedi AK Padovani A Berg D Garcia-Ruiz PJ. Benign versus malignant Parkinson disease: the unexpected silver lining of motor complications. J Neurol. (2020) 267:2949–60. 10.1007/s00415-020-09954-6 32488298

[18] Yoon SY Suh JH Yang SN Han K Kim YW. Association of physical activity, including amount and maintenance, with all-cause mortality in parkinson disease. JAMA Neurol. (2021) 78:1446–53. 10.1001/jamaneurol.2021.3926 34724534 PMC8561431

[19] Mak MKY Schwarz HB. Could exercise be the answer? Disease modification with long-term regular physical activity in Parkinson disease. Neurology. (2022) 98:303–4. 10.1212/WNL.0000000000013208 35022307

[20] Oveisgharan S Yu L Dawe RJ Bennett DA Buchman AS. Total daily physical activity and the risk of parkinsonism in community-dwelling older adults. J Gerontol A Biol Sci Med Sci. (2020) 75:702–11. 10.1093/gerona/glz111 31046115 PMC7328202

[21] Chen H Zhang SM Schwarzschild MA Hernan MA Ascherio A. Physical activity and the risk of Parkinson disease. Neurology. (2005) 64:664–9. 10.1212/01.WNL.0000151960.28687.93 15728289

[22] Xu Q Park Y Huang X Hollenbeck A Blair A Schatzkin A. Physical activities and future risk of Parkinson disease. Neurology. (2010) 75:341–8. 10.1212/WNL.0b013e3181ea1597 20660864 PMC2918886

[23] Yang F Lagerros YT Bellocco R Adami HO Fang F Pedersen NL. Physical activity and risk of Parkinson's disease in the Swedish National March Cohort. Brain. (2015) 138(Pt 2):269–75. 10.1093/brain/awu323 25410713

[24] Shih I Liew Z Krause N Ritz B. Lifetime occupational and leisure time physical activity and risk of Parkinson's disease. Parkinsonism Relat Disord. (2016) 28:112–7. 10.1016/j.parkreldis.2016.05.007 27177695 PMC4914446

[25] Belvisi D Pellicciari R Fabbrini G Tinazzi M Berardelli A Defazio G. Modifiable risk and protective factors in disease development, progression and clinical subtypes of Parkinson's disease: what do prospective studies suggest? Neurobiol Dis. (2020) 134:104671. 10.1016/j.nbd.2019.104671 31706021

[26] Sujkowski A Hong L Wessells RJ Todi SV. The protective role of exercise against age-related neurodegeneration. Ageing Res Rev. (2022) 74:101543. 10.1016/j.arr.2021.101543 34923167 PMC8761166

[27] Tipton CM. The history of “Exercise Is Medicine” in ancient civilizations. Adv Physiol Educ. (2014) 38:109–17. 10.1152/advan.00136.2013 25039081 PMC4056176

[28] Lau YS Patki G Das-Panja K Le WD Ahmad SO. Neuroprotective effects and mechanisms of exercise in a chronic mouse model of Parkinson's disease with moderate neurodegeneration. Eur J. Neurosci. (2011) 33:1264–74. 10.1111/j.1460-9568.2011.07626.x 21375602 PMC3079264

[29] Real CC Ferreira AF Chaves-Kirsten GP Torrão AS Pires RS Britto LR. BDNF receptor blockade hinders the beneficial effects of exercise in a rat model of Parkinson's disease. Neuroscience. (2013) 237:118–29. 10.1016/j.neuroscience.2013.01.060 23396085

[30] Binda KH Lillethorup TP Real CC Bærentzen SL Nielsen MN Orlowski D. Exercise protects synaptic density in a rat model of Parkinson's disease. Exp Neurol. (2021) 342:113741. 10.1016/j.expneurol.2021.113741 33965411

[31] Hirsch MA Iyer SS Sanjak M. P Exercise-induced neuroplasticity in human Parkinson's disease: what is the evidence telling us? Parkinsonism Relat Disord. (2016) 22(Suppl. 1):S78–81. 10.1016/j.parkreldis.2015.09.030 26439945

[32] Johansson ME Cameron IGM Van der Kolk NM de Vries NM Klimars E Toni I. Aerobic exercise alters brain function and structure in parkinson's disease: a randomized controlled trial. Ann Neurol. (2022) 91:203–16. 10.1002/ana.26291 34951063 PMC9306840

[33] Mahalakshmi B Maurya N Lee SD Bharath Kumar V. Possible neuroprotective mechanisms of physical exercise in neurodegeneration. Int J Mol Sci. (2020) 21:5895. 10.3390/ijms21165895 32824367 PMC7460620

[34] Ruiz-González D Hernández-Martínez A Valenzuela PL Morales JS Soriano-Maldonado A. Effects of physical exercise on plasma brain-derived neurotrophic factor in neurodegenerative disorders: a systematic review and meta-analysis of randomized controlled trials. Neurosci Biobehav Rev. (2021) 128:394–405. 10.1016/j.neubiorev.2021.05.025 34087277

[35] Kowiański P Lietzau G Czuba E Waśkow M Steliga A Moryś J. BDNF: a key factor with multipotent impact on brain signaling and synaptic plasticity. Cell Mol Neurobiol. (2018) 38:579–93. 10.1007/s10571-017-0510-4 28623429 PMC5835061

[36] Baydyuk M Xu B. BDNF signaling and survival of striatal neurons. Front Cell Neurosci. (2014) 8:254. 10.3389/fncel.2014.00254 25221473 PMC4147651

[37] Ellis T Boudreau JK DeAngelis TR Brown LE Cavanaugh JT Earhart GM. Barriers to exercise in people with Parkinson disease. Phys Ther. (2013) 93:628–36. 10.2522/ptj.20120279 23288910 PMC3641403

[38] Schootemeijer S van der Kolk NM Ellis T Mirelman A Nieuwboer A Nieuwhof F. Barriers and motivators to engage in exercise for persons with Parkinson's disease. J Parkinsons Dis. (2020) 10:1293–9. 10.3233/JPD-202247 32925106 PMC7739964

[39] Sokol LL Shapiro D Young MJ Wise AH Hadelsberg UP Kaufman Y. The Parkinson care advocate: integrating care delivery. Front Neurol. (2017) 8:364. 10.3389/fneur.2017.00364 28798721 PMC5529407

[40] Franzén E Johansson H Freidle M Ekman U Wallén MB Schalling E. The EXPANd trial: effects of exercise and exploring neuroplastic changes in people with Parkinson's disease: a study protocol for a double-blinded randomized controlled trial. BMC Neurol. (2019) 19:280. 10.1186/s12883-019-1520-2 31718583 PMC6849188

